# Use of two indicators for the socio-environmental risk analysis of Northern Mexico under three climate change scenarios

**DOI:** 10.1007/s11869-014-0286-3

**Published:** 2014-08-08

**Authors:** Armando López-Santos, Santos Martínez-Santiago

**Affiliations:** Unidad Regional Universitaria de Zonas Áridas, Universidad Autónoma Chapingo, Km. 40 Carretera Gómez Palacio-Chihuahua, Bermejillo, Dgo CP 35230 Mexico

**Keywords:** Dryland, Wind erosion, Downscaling, Drought, Adaptation

## Abstract

The aims of this study were to (1) find critical areas susceptible to the degradation of natural resources according to local erosion rates and aridity levels, which were used as environmental quality indicators, and (2) identify areas of risk associated with the presence of natural hazards according to three climate change scenarios defined for Mexico. The focus was the municipality of Lerdo, Durango (25.166° to 25.783° N and 103.333° to 103.983° W), which has dry temperate and very dry climates (BSohw and BWhw). From the Global Circulation Models, downscaling techniques for the dynamic modeling of environmental processes using climate data, historical information, and three regionalized climate change scenarios were applied to determine the impacts from laminar wind erosion rates (LWER) and aridity indices (AI). From the historic period to scenario A2 (ScA2, 2010–2039), regarding greenhouse gas emissions, the LWER was predicted to reach 147.2 t ha^−1^ year^−1^, representing a 0.5 m thickness over nearly 30 years and a change in the AI from 9.3 to 8.7. This trend represents an increase in drought for 70.8 % of the study area and could affect 90 % of the agricultural activities and approximately 80 % of the population living in the southeastern Lerdense territory.

## Introduction

The analysis of socio-environmental (SE) risk is used to study the causes of and damage by potential threats or hazards and undesired events related to climate variability as well as the consequences for vulnerable people, property, and ecosystems (IPCC 2012; Lim et al. 2005). For example, the likely impacts of changes in both rainfall and runoff on water availability pose critical problems globally; approximately 80 % of the world’s population (7.1 billion in 2013) reside in areas where the supply of fresh water is not secure (Howell et al. 2013; Poulsen 2013). Dryland areas cover approximately 45 % of the planet’s surface and support approximately 2 billion people, 33.8 % of the world’s population, who live in areas represented by climatic classes B and C (Al-Kaisi et al. 2012; Poulsen 2013). SE analyses of these areas have revealed critical situations in terms of the depression of local economies, which is often exacerbated by the impacts of drought on economic activities and natural resources (water, soil, flora, and fauna) and is expressed as processes of desertification in many cases. Mexico is no exception, especially in the northern territory (IPCC 2012; Rivera et al. 2007; Magaña et al. 2012).

To improve our understanding of the complex relationships among the climate system, ecosystems, and human activities, the scientific community has developed scenarios that provide plausible accounts of how key socioeconomic and technological areas and environmental conditions could be affected by greenhouse gases (GHG) emissions and climate change (Moss et al. 2010). However, the implementation of these scenarios at the local level, which has significance for vulnerability studies on adaptation to climate change, is a growing challenge (DOF 2012; Stavi and Lal 2014).

The future climatic scenarios produced by Magaña and Caetano (2007) present anomalies of rain and temperature; these scenarios are the result of numerous experiments based on the 23 Global Circulation Models (GCMs) proposed by the Intergovernmental Panel on Climate Change (IPCC). In fact, this work is the origin of the GHG emission lines (IPCC 2007; Conde et al. 2011). These future climatic scenarios have the following characteristics: *Scenario A1* assumes a very rapid rate of global economic growth, a doubling of the world population by mid-century, and the rapid introduction of new and more efficient technologies. It is divided into three sub-scenarios that reflect three alternative directions for technological change: intensive fossil fuels (A1FI), non-fossil energy (A1T), and a balance between the various energy sources (A1B); *Scenario B1* describes a convergent world with the same population as in A1 but with a more rapid evolution of economic structures toward a service and information economy; *Scenario A2* describes a very heterogeneous world with strong population growth, slow economic development, and slow technological change.

The assessment scenarios (A1B, A2, B2, and B1) of GHG emissions show an approximately 3 % reduction in annual rainfall, a nearly 1 °C increase in the annual average temperature, and a 6 °C temperature increase in the fall. The longest drought occurs during 2010–2039, particularly in the fragile dry lands located in northern Mexico (Monterroso et al. 2011; Conde et al. 2011; Magaña et al. 2012).

Many studies have demonstrated that reduced moisture and higher ambient temperatures would result in an increased susceptibility of the soil to the effects of environmental factors, especially wind associated with increased dust storms and aridity (López-Santos et al. 2013; Zhang et al. 2013); for example, López-Santos et al. (2013)), using a technique of reducing the dynamic range in the ScA2 for Gomez Palacio, Durango, Mexico, noted that the impacts of drought could affect up to 63 % of the local territory by 2030 and cause soil losses of 151.4 t ha^−1^ year^−1^ due to wind erosion.

Lerdo is a municipality located in the driest area of northwestern Durango State. Including the neighboring municipalities of Torreon and Gomez Palacio, almost 70 % of the regional population (1.8 million) is concentrated in the area. According to INECC-SEMARNAT (2012), two of the main features of this region are as follows: (1) there are two key water systems for the region, i.e., the Nazas-Torreon (79 %) and Aguanaval (20.1 %) watersheds, and (2) only 24 % of the territory is suitable for continuous mechanized agriculture. Accordingly, the aims of this study were to (1) find critical areas that are susceptible to the degradation of natural resources based on the erosion rates and aridity levels, which are used as indicators of environmental quality, and (2) identify areas of risk associated with the occurrence of natural hazards based on three climate change scenarios defined for Mexico.

## Methodology

### Study area, characterization, and location

**Fig. 1:**
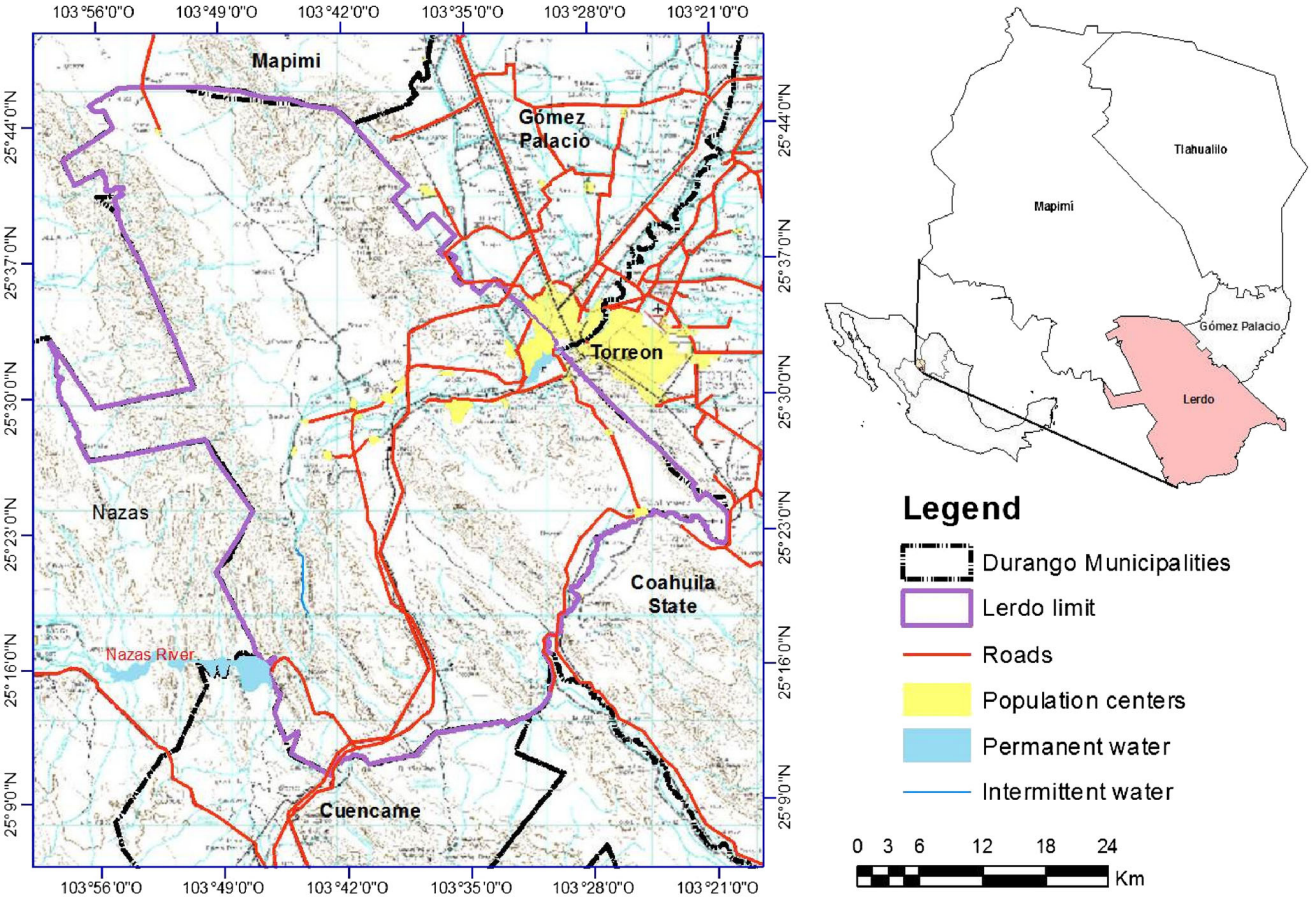
Geographical location and topographic features of Lerdo, Durango, Mexico

Lerdo is one of 39 municipalities of Durango State, which has a territorial extent of 2,106 km^2^ and is located in a region (25.166° to 25.783° N and 103.333° to 103.983° W) that stretches across a climatic gradient of dry temperate to very dry (BSohw, BWhw) from south to north. The mean annual temperature varies between 14 and 22 °C, and the mean annual rainfall is approximately 300 mm (INEGI 2013) (Fig. 1).

### Baseline year and environmental quality indices

The baseline year, or reference year, for the study was 2010. The environmental quality index (EQI) chosen to assess the impacts of climate change over 30 years (2010–2039) were soil loss in layer form by the action of wind (laminar wind erosion rates, LWER) and drought expressed as the aridity index (AI), both of which are directly related to future changes in temperature and rainfall, as described by Magaña et al. (2012) for northern and northeastern Mexico.

### Modeling of the EQI

The calculation and modeling of the LWER is based on the methodology proposed by SEDESOL-INE (1998) for studies of ecological land management (SELM) and has been used by López-Santos et al. (2013) to model local impacts of climate change in northern Mexico according to four types of probable soil loss in ton per hectare per year: (1) light, <10; (2) moderate, 10–50; (3) high, 50–200; and (4) high, >200. This classification is set by the availability of environmental moisture following rainfall. In addition, AI was calculated by De Martonne’s index, which has been used for the drylands of northern Mexico in several studies (Salinas-Zavala et al. 1998; López-Santos et al. 2013), to indicate drought (see “Aridity index as an indicator of drought” section).

### Inputs for modeling

The inputs for modeling, in addition to those related to the physical–biotic environment, such as topography, soil, and vegetation (see description below), were the base year historical weather data from the National Weather Service. To evaluate the potential changes in the EQI for the 2010–2039 impact scenario, the regionalized climate change projections (metadata) for Mexico (SEMARNAT-INECC 2011) generated from the downscaling results of the GCM presented in the Fourth Assessment Report of the IPCC (2007) were used.

#### Procedure for determining the LWER

As an index of environmental quality, the LWER (t ha^−1^ year^−1^) represents the magnitude of soil loss by wind action, which is theoretically incorporated as a contaminant at different heights in the atmosphere. Soil particles suspended in the lowest layer of the atmosphere have a direct effect on human health because people breathe the contaminated air; thus, human populations in urban and rural areas are affected according to their degree of exposure.

In the procedure for determining the LWER for the base year (2010), according to the climate that was previously described (i.e., BSohw and BWhw), it was assumed that the dominant environmental factor is the wind; thus, wind was used in the following equation: (Eq. 1)

1 $$ \mathrm{LWER}=\mathrm{IAVIE}\times \mathrm{CATEX}\times \mathrm{CAUSO} $$

where CATEX (Spanish acronym) is an index related to soil properties, CAUSO (Spanish acronym) is an index defined by the different land uses, CAERO (Spanish acronym) is an index of the degree of susceptibility to soil erosion, and CATOP (Spanish acronym) refers to an index that describes the topographical conditions.

The rationale for calculating erosion rates is related to the availability of humidity due to the presence of rain >10 mm; thus, the LWER index of wind aggressiveness (IAVIE, Spanish acronym) was calculated according to the method described by Lopez-Santos et al. (2013) using the following equation: (Eq. 2).

2 $$ \mathrm{IAVIE}=160.8252-0.7660\times \left(\mathrm{GROPE}\right) $$

where GROPE is defined as the number of days per year with suitable water availability and favorable conditions for the establishment of a crop; it was proposed by Ortiz-Solorio (1987) and has been used in several studies (López-Santos et al. 2013; Monterroso et al. 2011). It is calculated as (Eq. 3).

3 $$ \mathrm{GROPE}=0.2408\times \left(\mathrm{MAR}\right)-0.0000372\times {\left(\mathrm{MAR}\right)}^2-33.1019 $$

#### Source and management of climatic data

**Fig. 2:**
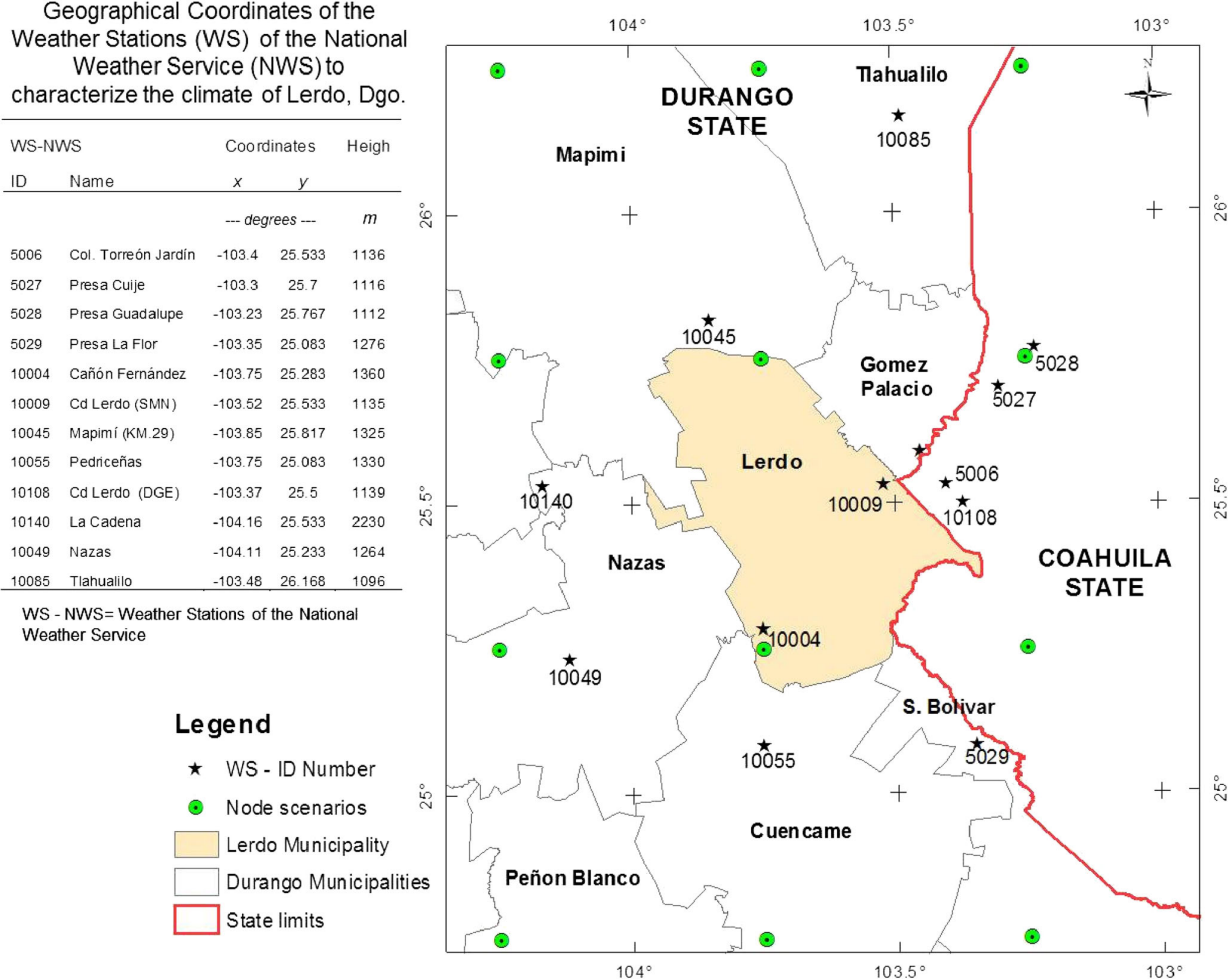
Integration of selected WS and geographical locations

The data from 12 weather stations (WS) of the National Weather Service that are located in the northern region of the municipality were used. All of the stations are within a radius of approximately 65 km. Four WS were located in the state of Coahuila (WS Id numbers: 5006, 5027, 5028, and 5029), and the other nine were in Durango State. The metadata for the future scenarios are presented for the area of coverage (Fig. 2).

### Indices related to edaphic properties and land use: CATEX and CAUSO

The estimated values for the CATEX and CAUSO indices for the municipality of Lerdo were created by manipulating the attribute tables of the vector sets for Soil Science subjects and Land Use and Vegetation (LUV), respectively. The CAUSO index, which is associated with the distribution and abundance of vegetation, was estimated based on the identification of four vegetation groups and on the proportions of land dedicated to agricultural use and under irrigation (Table 1).

**Table 1:** CAUSO and CATEX indices used for modeling

CATEX	TCPP	C-INEGI, 2007	CAUSO	Category	Clave^a^
3.5	1	Coarse	0.70	C1	T
1.75	2	Medium	0.20	C2	R
1.85	3	Fine	0.15	C3	Mdm, Mdr, Ch, Vg
0.87	SPB	SPB	0.30	C4	P, Vh
NA	NA	NA	NA	NA	Cau, Ur, H2O

*TCPP* textural class and physical phase, *C-INEGI, 2007* textural class according to INEGI, 2007 (INEGI 2007a), *SPB* stony phase or burden, *NA* not applicable, *T* rainfed agriculture, *R* irrigated agriculture, *Mdm* microphyllous scrubland, *Mdr* rosette scrubland, *Ch* chaparral, *Vg* gallery vegetation, *P* grassland, *Vh* hydrophyllum vegetation, *Cau* riverbed, *Ur* urban area, *H*_*2*_ *O* water body

^a^Cartographic clave used for vegetation maps from INEGI editions 1975 and 2009

### Metadata climate scenarios (2010–2039) to determine LWER

To assess changes in climate, the metadata portal website INE-SEMARNAT, which is now available at the Atmospheric Sciences Center of UNAM, was downloaded (http://atlasclimatico.unam.mx/atlas/kml/) to acquire the mean annual precipitation (MAR), temperature (MAT), and three future roadmap scenarios (A2, A1B, and B1) for the period 2010–2039 (Table 2).

**Table 2:** HMAR, HMAT, and anomalies regionalized for Mexico for three future scenarios during 2010–2039

Clave	WS name	Coordinates			MAR	Anomaly			MAT	Anomaly		
		*x*	*y*	*z*	Hist	A2	A1B	B1	Hist	A2	A1B	B1
		Dg		m	mm	%			°C			
5006	Torreón Jardín	−103.4	25.533	1,136	256.2	−2.55	−3.08	−2.79	22.0	0.86	0.926	0.838
5027	Presa Cuije	−103.3	25.7	1,116	194.0	−3.32	−3.08	−2.79	21.9	0.86	0.926	0.838
5028	Presa Guadalupe	−103.23	25.767	1,112	203.3	−3.32	−3.08	−2.79	21.5	0.86	0.926	0.838
5029	Presa La Flor	−103.35	25.083	1,276	280.3	−2.55	−2.87	−2.94	20.9	0.856	0.929	0.836
10004	Cañón Fernández	−103.75	25.283	1,360	320.4	−3.98	−3.94	−3.69	22.1	0.867	0.935	0.835
10009	Cd Lerdo (SMN)	−103.52	25.533	1,135	286.6	−2.98	−2.95	−2.24	21	0.851	0.926	0.823
10045	Mapimí (KM.29)	−103.85	25.817	1,325	320.1	−2.98	−2.95	−2.24	19.3	0.851	0.926	0.823
10055	Pedriceñas	−103.75	25.083	1,330	403.6	−3.98	−3.94	−3.69	19.6	0.867	0.935	0.835
10108	Cd Lerdo (DGE)	−103.37	25.5	1,139	270.1	−2.55	−2.87	−2.94	21	0.856	0.929	0.836
10140	La Cadena	−104.16	25.533	2,230	274.2	−3.76	−2.84	−2.73	21.2	0.865	0.973	0.834
10049	Nazas	−104.11	25.233	1,264	348.9	−2.81	−2.95	−2.61	20.3	0.883	0.958	0.85
10085	Tlahualilo	−103.48	26.168	1,096	266.4	−1.64	−2.12	−2.74	20.6	0.861	0.92	0.827
			Mean	1,293	285.3	−3.04	−3.06	−2.85	20.95	0.861	0.934	0.834
			Stdev	311	58.1	0.69	0.49	0.45	0.89	0.009	0.016	0.007

*HMAR* historic mean annual rain, *HMAT* historic mean annual temperature, *WS* weather station, *Stdev* standard deviation, *Dg* decimal degrees, *x* west longitude, *y* north latitude, *z* height, *m* meters

### Aridity index as an indicator of drought

De Martonne’s AI was used for characterizing climate and indicating drought (Salinas-Zavala et al. 1998). For this study, the following expression was applied: (Eq. 4).

4 $$ \mathrm{AI}=\mathrm{MAR}/\left(10 + \mathrm{MAT}\right) $$

AI is the aridity index, which is a dimensionless value between 0 and >60. MAR is the mean annual rainfall from historical records and ScA2. MAT is the mean annual temperature from historical records and ScA2, and 10 is a constant value derived from De Martonne’s model. The classification has six categories each index range: (i) desert or hyperarid (0–5), (ii) semi-desert or arid (5–10), (iii) semiarid Mediterranean type (10–20), (iv) subhumid (20–30), (v) wet (50–60), and (vi) perwet (>60) (González 2012).

### Geostatistical analysis and impact assessment

The impact analysis of future climatic variability in terms of rainfall and temperature began with the creation of a projected layer (shp) according to the locations (*x*, *y*) for each WS. The data were interpolated for all of the variables included in the study (zi) using an inverse distance-weighted (IDW) method in ArcMap 10.1® (ESRI, Redlands, CA, USA); the same method has been used in similar studies (Karaca 2012; López-Santos et al. 2013). The first product of this process was the creation of a raster layer adjusted to the maximum and minimum extreme values. The second product was the change in the properties of the statistical and pixelated raster images for the three classes and was created by calculating the changes from the historical or current data to the future scenarios. Next, the classified raster images were converted to a vector format, which made it possible to determine the surface terms that were most impacted by climate change.

## Results and discussion

From the historical values and anomalies shown in Table 2, the values of MAR and MAT for the A2, A1B, and B1 future scenarios were determined for an approximately 30-year period (2010–2039). The anomalies of the MAR, which were negative, were obtained by subtracting the historical value from the equivalent value in millimeter, while the MAT (in °C) was calculated by direct addition. The anomalies for both variables (MAR and MAT) were very similar among the three future scenarios, as described by Magaña and Caetano (2007). Hence, the impact analysis focused only on the ScA2 scenario.

### Analysis of the impact on MAR

When comparing the behavior of the HMAR and what would be expected for the mean annual rainfall in an aggressive environment such as the ScA2, it appears that the impact of these anomalies on the spatial distribution of annual rainfall leads to an improvement in water availability, considering that the range (low-high) changes from 255–314 to 258–347 mm, representing increases of 3 and 33 mm for the lower and upper limits, respectively, which equates to increases of 1.16 and 9.51 %, respectively (Table 3).

**Table 3:** Impact analysis on MAR in the scenario A2

Range	Variable		Impact	
	HMAR	PMA_A2_	AC	RI
	mm			%
Low	255	258	3	1.16
High	314	347	33	9.51
Mean	284	302	18	5.95

*HMAR* historic mean annual rainfall, *MAR*_A2_ mean annual rainfall under scenario A2, *AC* absolute change (HMAR − MAR_A2_), *RI* relative impact

**Fig. 3:**
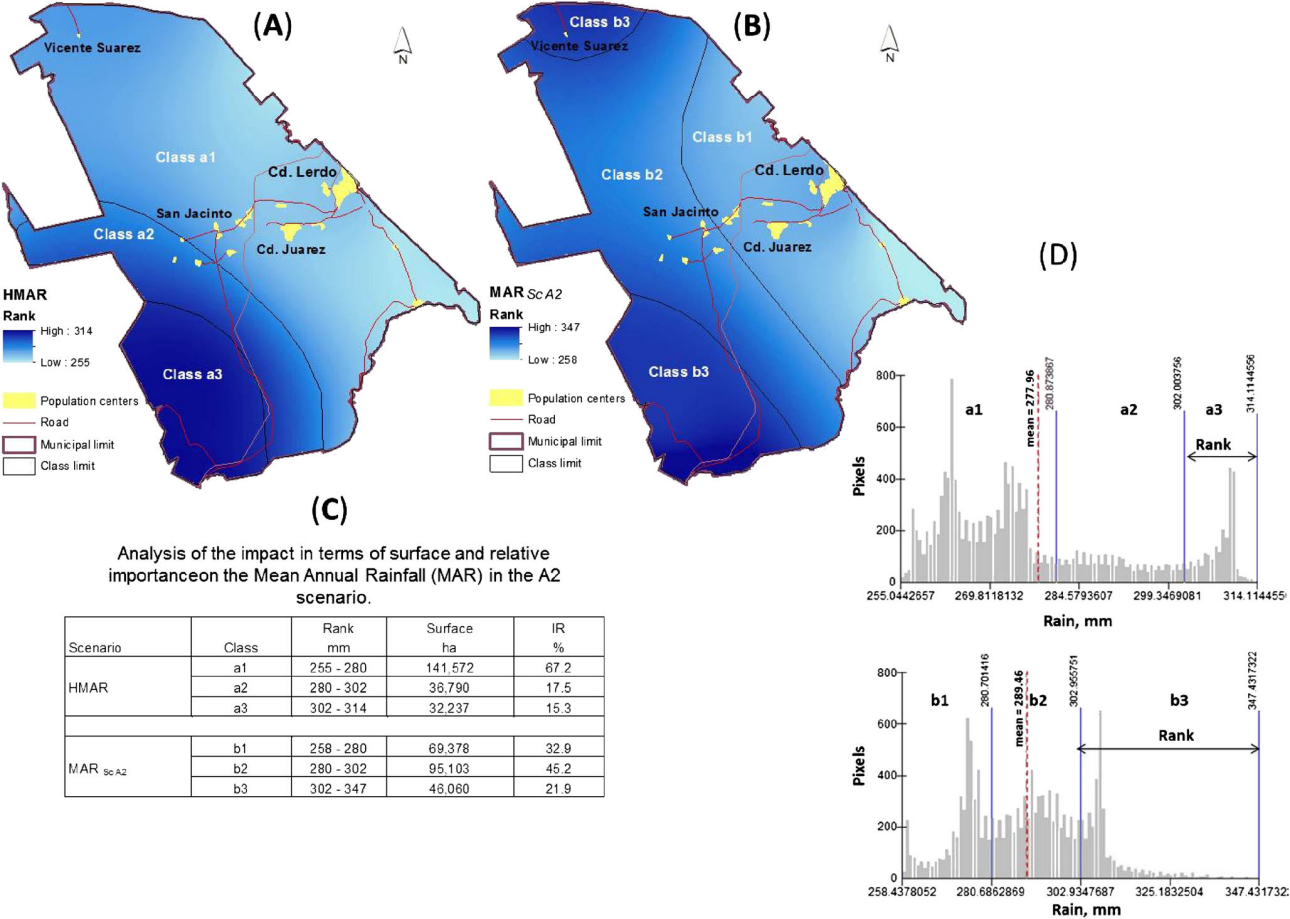
Spatial distribution of the MAR and impact of scenario A2

To complement the analysis of the changes in the MAR between the historical and ScA2 levels, additional digitally processed raster images for the distribution of MAR, including both the historical and ScA2 values, were produced to compare approximately equal ranks; the data were reclassified into three classes for each case, and the surface areas and distributions of each class were calculated (Fig. 3). The results of this process (reclassification) were accompanied by a statistical analysis of the distribution and concentration of pictorial values (pixels) of each of the six ranges of rain: three for the historical data and three for the ScA2 data. These results reveal, for example, that although the area of class 3 increased in terms of the total area between the historical data (15.3 %; 32,237 ha) and ScA2 (21.9 %; 46,060 ha), the highest pixel density under ScA2 was concentrated at the lower limit of the range (302 mm), while the pixel density of the upper range (347 mm) was much lower (Fig. 3d).

### Calculation and zoning GROPE

According to the above-described changes in MAR, the mean GROPE rate under ScA2 increased by 8.03 % from the historical mean, while the low-high limits changed from 26.9–40.2 for the historical scenario to 26.6–46.1 for scenario A2 (Table 4).

**Table 4:** Impact analysis for the GROPE index as a consequence of MAR changes

Range	Variable		Impact	
	HGROPE	GROPE_A2_	AC	RI
	Index			%
Low	26.9	26.6	0.3	1.11
High	40.2	46.1	5.9	14.67
Mean	33.6	36.3	2.7	8.03

*HGROPE* historic growing season, *GROPE*_A2_ growing season for scenario A2, *AC* absolute change (HGROPE − GROPE_A2_), *RI* relative impact (AC/HGROPE)

**Fig. 4:**
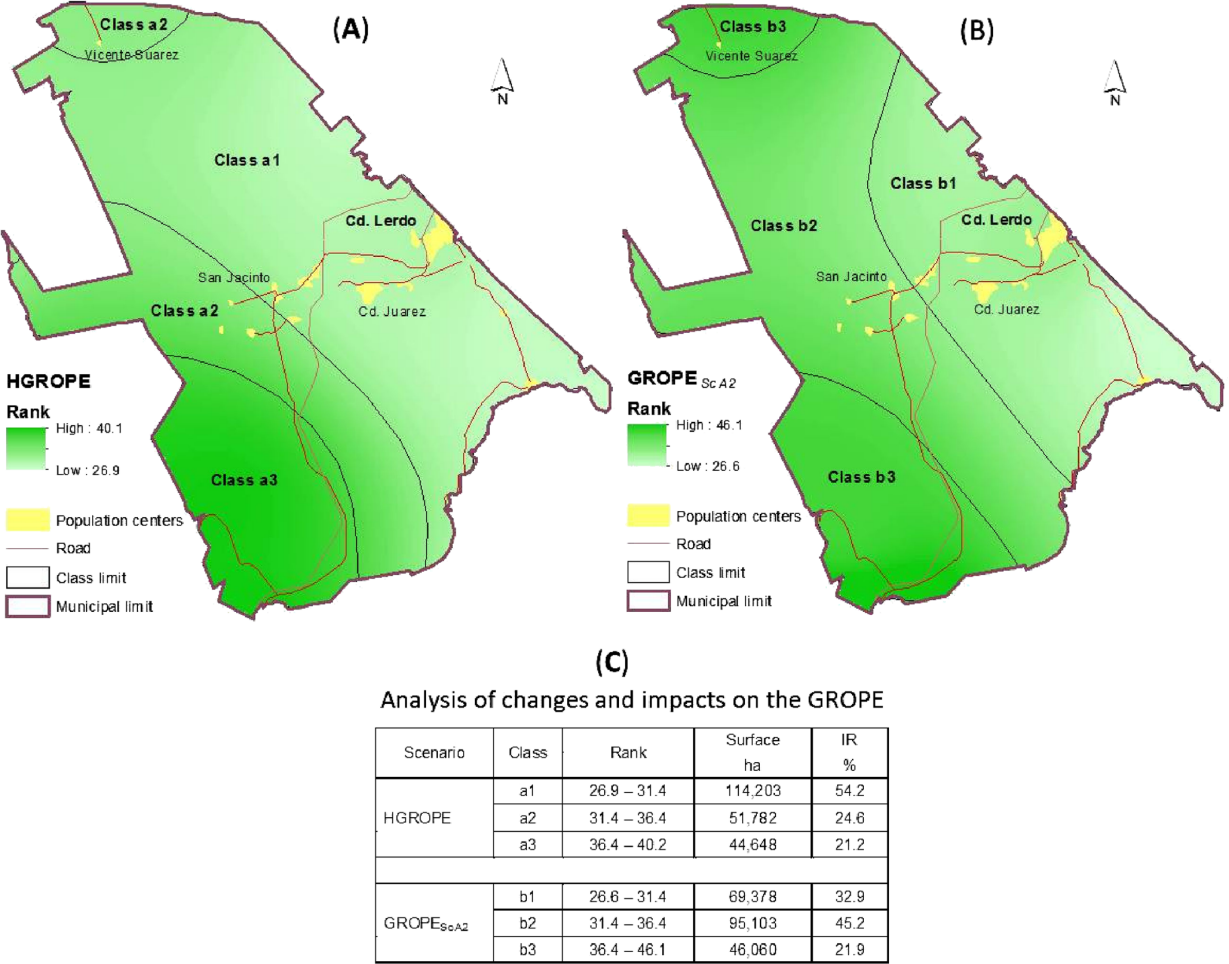
Spatial distribution of *GROPE* for the historical data and scenario A2

The existence of a relationship between the MAR and the GROPE index (Eq. 2) is clear because the degree to which the MAR affected this index somewhat changed proportionally to the change in MAR. However, it is not easy to determine the magnitude of the impacts of this signal considering that the limits of the range of GROPE (Fig. 4) are interpreted as follows:

The lower limit of the range for the GROPE index changes from 26.9 to 26.3 under ScA2, which is an absolute difference of −0.3; this trend indicates less rainfall in terms of the annual mean conditions and would presumably reduce the viability for establishing a crop. Under this premise, the change in GROPE is expected to affect an area of 69.378 ha, equivalent to 32.9 % of the municipal total, the Sierra del Sarnoso, and the surrounding rural populations of Luz, La Mina, the main city (Lerdo), the valley of León Guzman, Juarez, and Nazareno. However, the upper limit of the range for the *GROPE* index increased from 40.2 to 46.1 under ScA2, an absolute difference of 5.9, which is apparently beneficial because this change can be interpreted as an increase in the probability of the successful establishment of a crop that can even thrive beyond germination. However, the benefit associated with this increase in the GROPE index affects a much smaller area due to the way in which the changes in MAR are distributed, as described in the previous section (Fig. 3) for the statistics of the pictorial values of the raster image used for analyzing this phenomenon.

### Calculation and distribution of the edaphic index (CATEX)

**Fig. 5:**
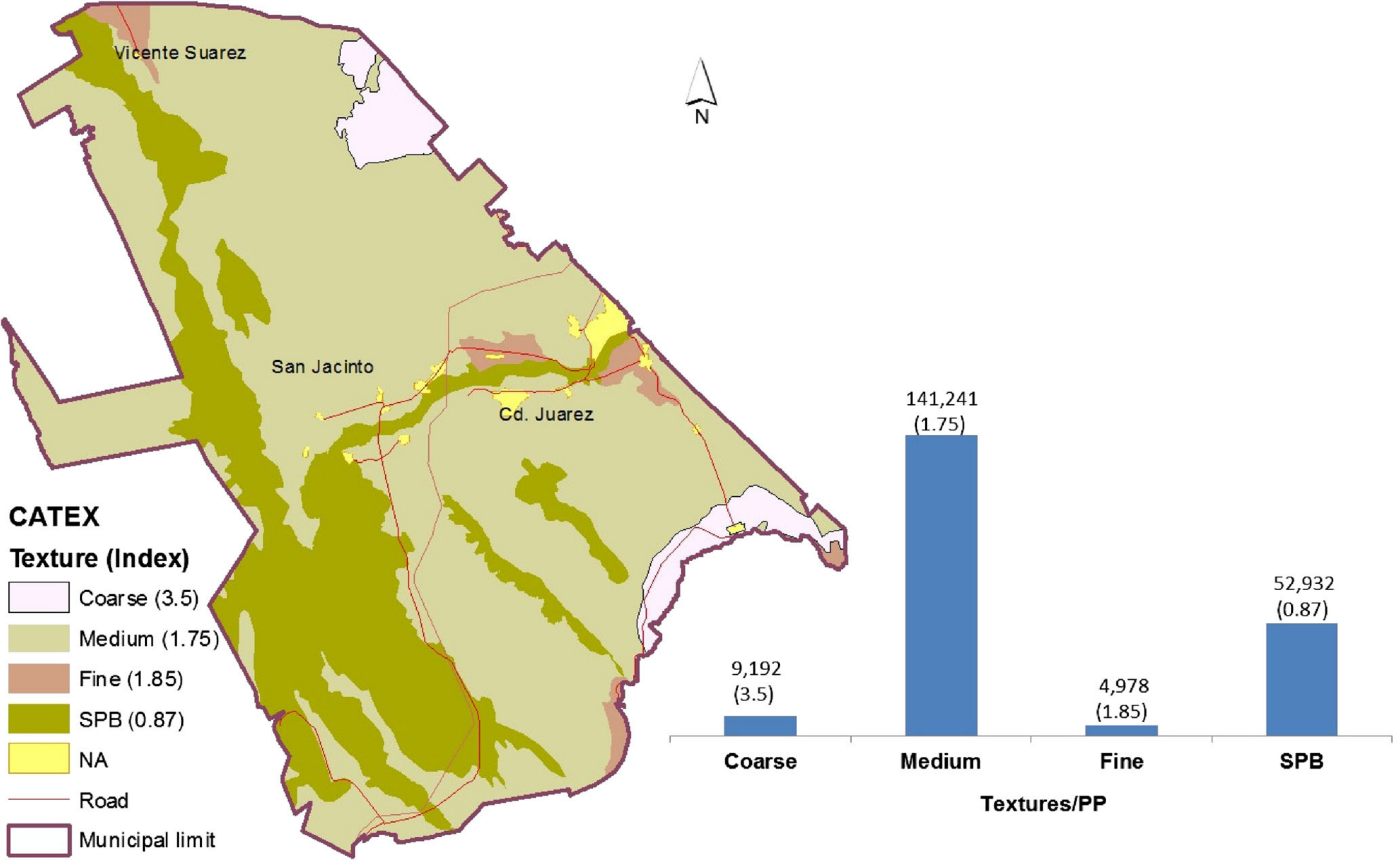
Spatial distribution of the CATEX index and relative importance

The CATEX index, as already indicated, was determined based on the soil textural classification for the three groups and the contents of stony and physical phases using the integrated set of vector data from the INEGI (2007b) Soil Science of Torreon coverage (Code: G13-09) in which the soil units are classified according to the World Reference Base for Soil Resources [WRB, (FAO-ISRIC-ISSS 1998)]. Accordingly, most of the municipal territory (72.2 %) contains coarse and medium soil textures, which are defined as classes 1 and 2, respectively. Additionally, surface outcrops with coarse sand (>2 mm diameter) and materials classified as gravel, FPG, were identified (Fig. 5).

In addition to the distribution described above, the identification of textural groups and physical phases revealed that medium-textured soils (class 2) covered 67.79 % of the municipal territory, followed by stony soils (SPB; 25.41 %), which are located to the west and southwest of the city in the locations described above. In contrast, soils with either coarse or fine textures with relative importance values of 4.41 and 2.39 %, respectively, are only located in small areas of the southeast and northeast (Fig. 5).

### CAUSO index and changes up to 2039

**Fig. 6:**
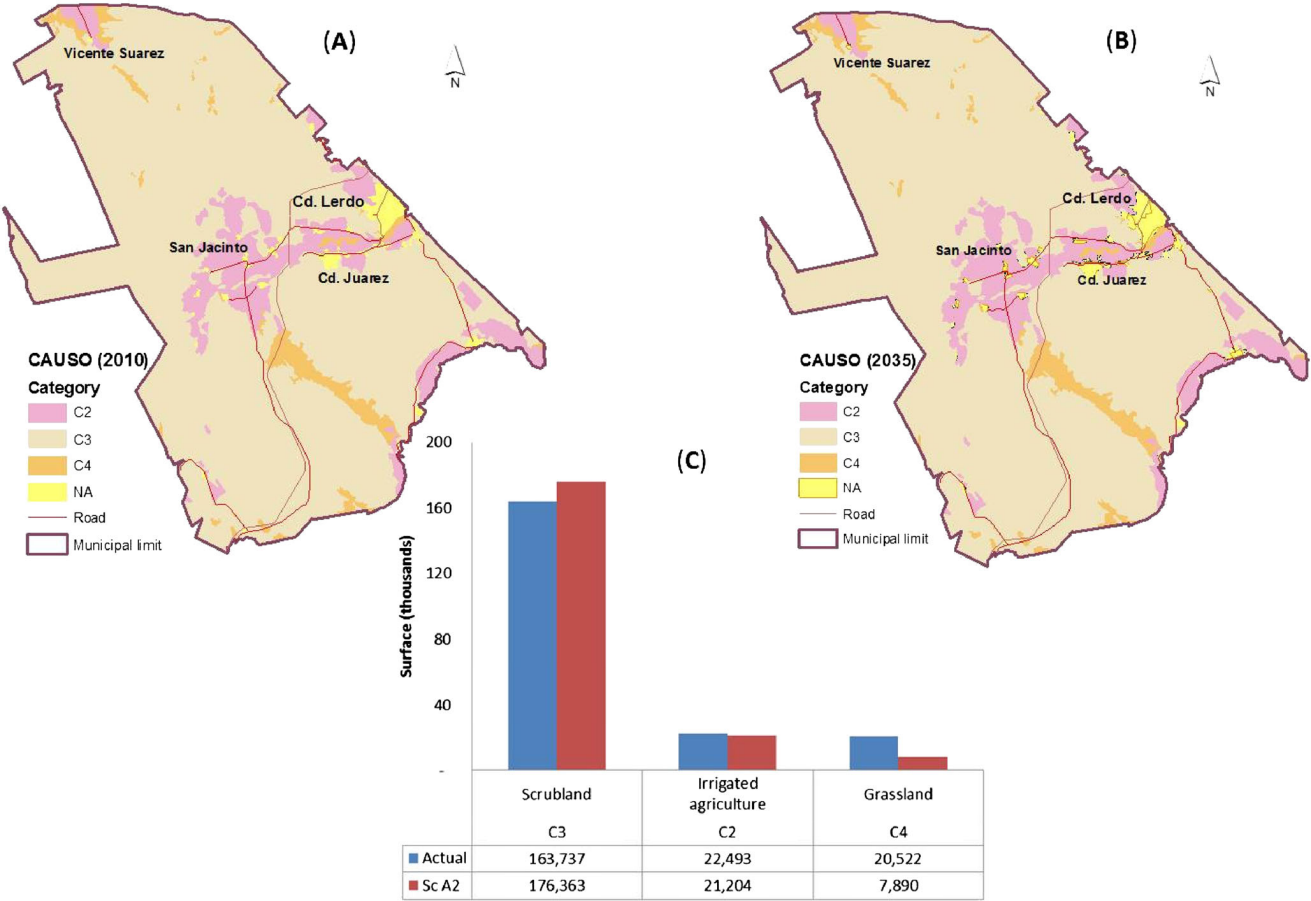
Changes in the CAUSO index for three categories of land use

The CAUSO index and distribution of various land uses for a period of slightly over 30 years (2010–2039) are based on estimates from the Urban Development Plan [UDP, (SEMARNAT 2013)]. In the municipality of Lerdo, the distribution of the C4 category (Fig. 6) will be affected drastically in terms of grassland vegetation and halophyte vegetation located in small valleys to the north within the area that contains Vicente Suarez and in low areas in the south that are located between Cañon de Fernandez and Sierra de España.

### EQI for the historical records and scenario A2

The defined EQI (LWER and IA) used for determining the magnitude of the impact of climate change on the municipality of Lerdo is based on the calculation of anomalies of rainfall and temperature in the future under ScA2 (Table 2) and on the analysis of their geographical redistribution; the results are presented below.

#### Estimation and redistribution of LWER

The LWER calculated for the town of Lerdo was 17–147 t ha^−1^ year^−1^ and is in agreement with the ranges defined in the literature (SEDESOL-INE 1989). Of the total municipal area (136,694.4 ha), 65.2 % experiences a light laminar wind erosion of 23–51 t ha^−1^ year^−1^, whereas 12.2 % of the area (5769.3 ha) experiences a moderate to high LWER, at 51–76 to 76–147 t ha^−1^ year^−1^ (Fig. 8a). Within the context of ScA2, the results indicate minor changes in the wind erosion rate. The historic LWER was 17–147 compared with 16.8–147.2 for ScA2; thus, absolute and relative differences of 1.17 and 0.14 %, respectively, occur between the lower and upper limits (Table 5).

**Table 5:** Analysis of changes in the LWER between the historical data and scenario A2

Range	Variable		Impact	
	HLWER	LWER_A2_	AC	RI
	t ha^−1^ year^−1^			%
Low	17	16.8	0.2	1.17
High	147	147.2	0.2	0.14
Mean	82	82	0	0.00

*HLWER* historic laminar wind erosion rate, *LWE*_A2_ laminar wind erosion for Scenario A2, *AC* absolute change (HLWER − LWE_A2_), *RI* relative importance (AC/HEEL)

**Fig. 7:**
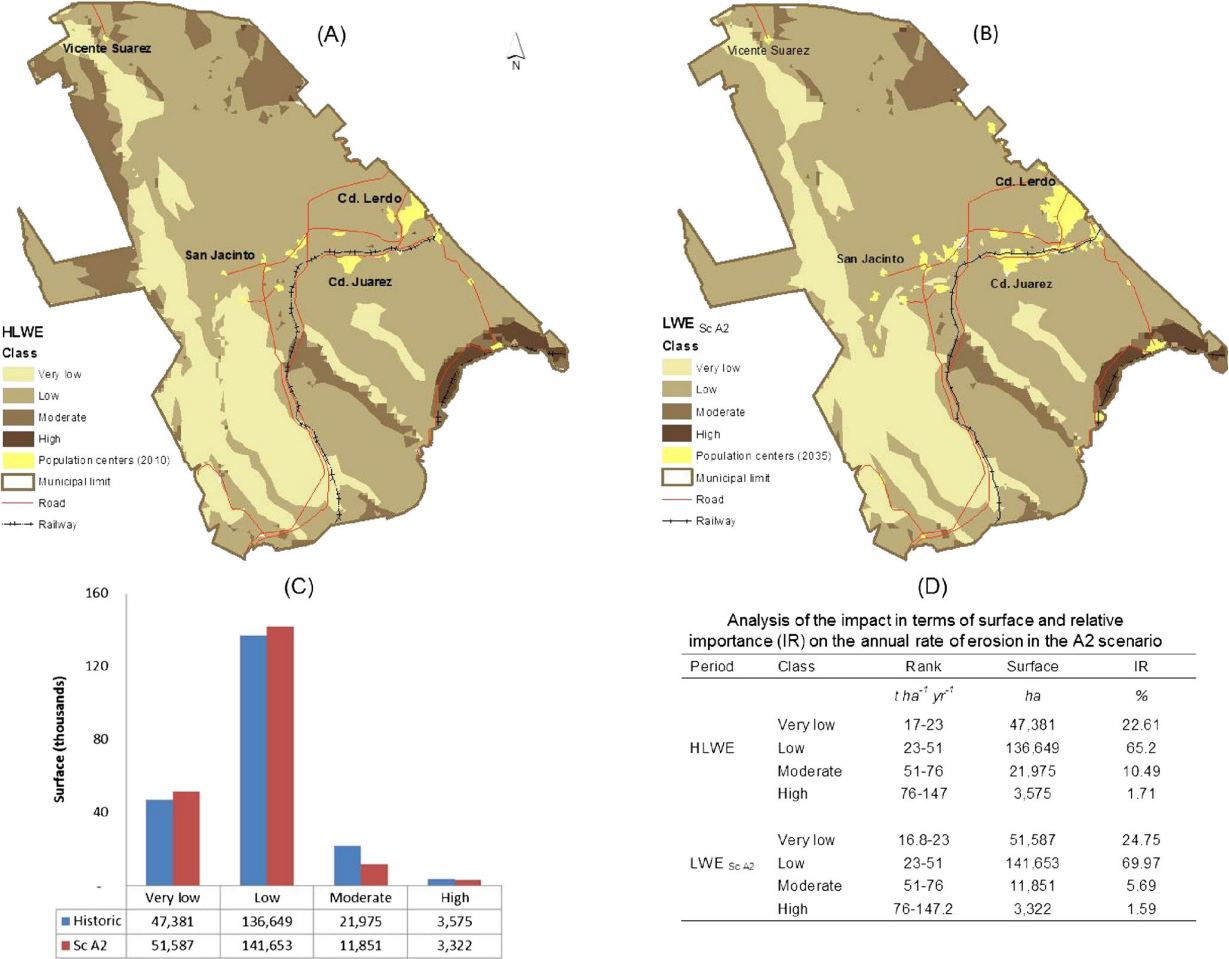
Spatial distributions of historic laminar wind erosion (HLWER) and LWER under ScA2

However, the impact analysis indicates that the small changes in the rate of the laminar wind erosion (±0.2 t ha^−1^ year^−1^), in terms of the surface area that is affected, create additional future problems by decreasing the moisture that is supplied by rain. Thus, it would be expected that the wind has an impact on an area larger than that observed from the historical data. For example, in the cases where areas are classified as having very light erosion (16.8–23 t ha^−1^ year^−1^) and light erosion (23–51 t ha^−1^ year^−1^), the extent of the affected area increases by 11.69 % between the historical data and scenario A2, ranging from 47.381 to 51.587 ha for the first (very light) class and from 136.649 to 141.653 ha for the second (light) class (Fig. 7d).

Additionally, it is important to mention that although the increase in light erosion could be a matter of little concern in the future scenario under consideration, the truth is that the likely impact of less rain and higher temperatures is the tendency for the area of this class to increase. The majority of the surface historically experienced below average light erosion, while under ScA2, the majority of the region experiences above average light erosion. The moderate to high classes of laminar wind erosion in the northeast are spatially distributed to the north of the municipality where the Sierras del Sarnoso of Mapimí are located and in small areas to the north in the agricultural valley of Vicente Suarez. To the west of the municipality, a critical area stretches across (north to south) the Sierra del Rosario; areas classified with high rates of erosion are located on the east side of the Sierra España, Villa Nazareno (Fig. 7).

#### Analysis of changes in AI

Considering the relationship that involves the model proposed by De Martonne (Eq. 4) for the variables considered in this study (MAR and MAT), i.e., the decrease in rainfall and increase in temperature as described above, it is expected that the current levels of AI are negatively impacted by the future adverse scenarios. Specifically, its manifestation may be in the form of greater environmental deterioration marked by increased aridity. Furthermore, the magnitude and distribution of the impact on the territory is highly important for planning natural resource management. It is therefore necessary information for developing strategies aimed at preventing the further deterioration of natural resources in the present and future. According to the calculations based on the anomalies already described for MAR and MAT (Table 2), the aridity index for the town of Lerdo changes from the historical mean of 9.3 to 8.7 under ScA2; thus, the change in the mean impact of this index in the future is estimated to be 0.53 ± 0.16 (Table 6).

**Table 6:** Estimated changes in the aridity index from historical conditions to Scenario A2

WS-NWS		Coordinates		Variable		
Clave	Name	*x*	*y*	HIA	IA_A2_	AC
		Dg		Index		
5006	Torreón Jardín	−103.400	25.533	8.0	7.6	0.41
5027	Presa Cuije	−103.300	25.700	6.1	5.7	0.36
5028	Presa Guadalupe	−103.230	25.767	6.5	6.1	0.38
5029	Presa La Flor	−103.350	25.083	9.1	8.6	0.47
10004	Cañón de Fernández	−103.750	25.283	10.0	9.3	0.65
10009	Cd. Lerdo (SMN)	−103.520	25.533	9.2	8.7	0.52
10045	Mapimí (KM.29)	−103.850	25.817	10.9	10.3	0.62
10055	Pedriceña	−103.750	25.083	13.6	12.7	0.91
10108	Cd. Lerdo (DGE)	−103.370	25.500	8.7	8.3	0.45
10140	La Cadena	−104.167	25.533	8.8	8.2	0.56
10049	Nazas	−104.117	25.233	11.5	10.9	0.64
10085	Tlahualilo	−103.483	26.168	8.7	8.3	0.38
			Mean	9.3	8.7	0.53
			Stdev	2.1	1.9	0.16

*Dg* decimal degrees, *AC* absolute change (HIA − IA_A2_), S*tdev* standard deviation

**Fig. 8:**
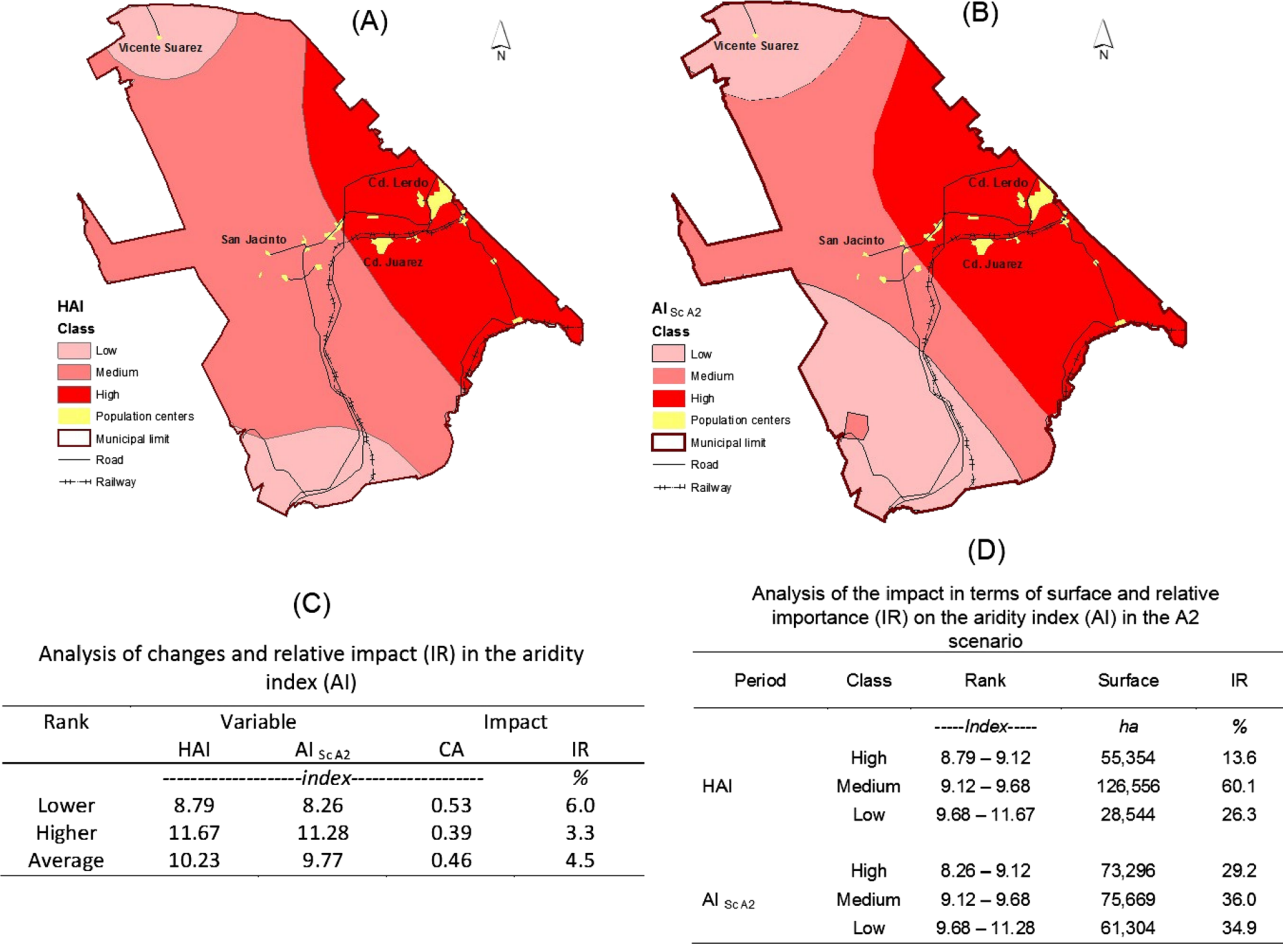
Spatial distribution of the aridity index (AI) for the historical data (**a**) and scenario A2 (**b**) based on the analysis of the absolute change (**c**) and relative impact (**d**) for the three classes of aridity

The absolute changes calculated in terms of territorial coverage (Fig. 8) that would be affected by an adverse future scenario (ScA2) indicate that areas classified as experiencing the two highest classes of aridity would total 148.956, which is equivalent to 70.8 % of the municipal area. Of these two classes, the area of the first class (high aridity) increases by 1.3 times that calculated for the historic area, while the surface area classified as experiencing moderate aridity decreases by 24.1 % from 126.566 to 75.669 ha, which represents a 1.6-fold decrease (Fig. 8d).

The spatial distribution of changes in the AI (Fig. 8a, b) indicates that the area of high AI extends from east to west through the center of the village where the municipal seat is located in the agricultural valley of León Guzman and to the Gum Ciudad Juarez area, where the majority of the vegetables consumed in the Laguna region are produced. It is also important to note that the lower and upper limits of the ranges change in absolute terms between the historical conditions (8.79–11.67) and scenario A2 (8.26–11.28), representing absolute differences of 0.53 and 0.39, respectively (Fig. 8c).

### Analysis of environmental quality and potential impacts

Environmental quality is a term associated with several aspects of “comfort,” both for people and for biological systems, and depends primarily on the availability of water, air quality, and temperature (Buchholz et al. 2014; Harlan et al. 2014; Karaca 2012). Water that falls as rain becomes an important regulator of temperature and, in turn, is related to extreme events, such as drought and dust storms (Al-Kaisi et al. 2012; Zhang et al. 2012). The recurrence of drought in recent years, coupled with poor natural resource management practices, has exacerbated environmental degradation globally (Howell et al. 2013; Stavi and Lal 2014; Poulsen 2013). Arid and semiarid regions are likely to experience an increase in temperature and decrease in rainfall (García-Páez and Cruz-Medina 2009; Magaña et al. 2012; Rivera et al. 2007), especially at the latitudes where Lerdo is located. Although the calculation used is based on only a slight increase in the mean temperature of 1 °C and a precipitation decrease of approximately 3 %, the actual future changes may be larger. For example, Magaña et al. (2012) reported that in northern Mexico, the average temperature at the end of the twenty-first century could increase 3.5 °C (±0.6) in the future under ScA2. The driest months (March, April, and May) could increase by 7 °C, while the annual rainfall could decrease by up to 5 %.

**Fig. 9:**
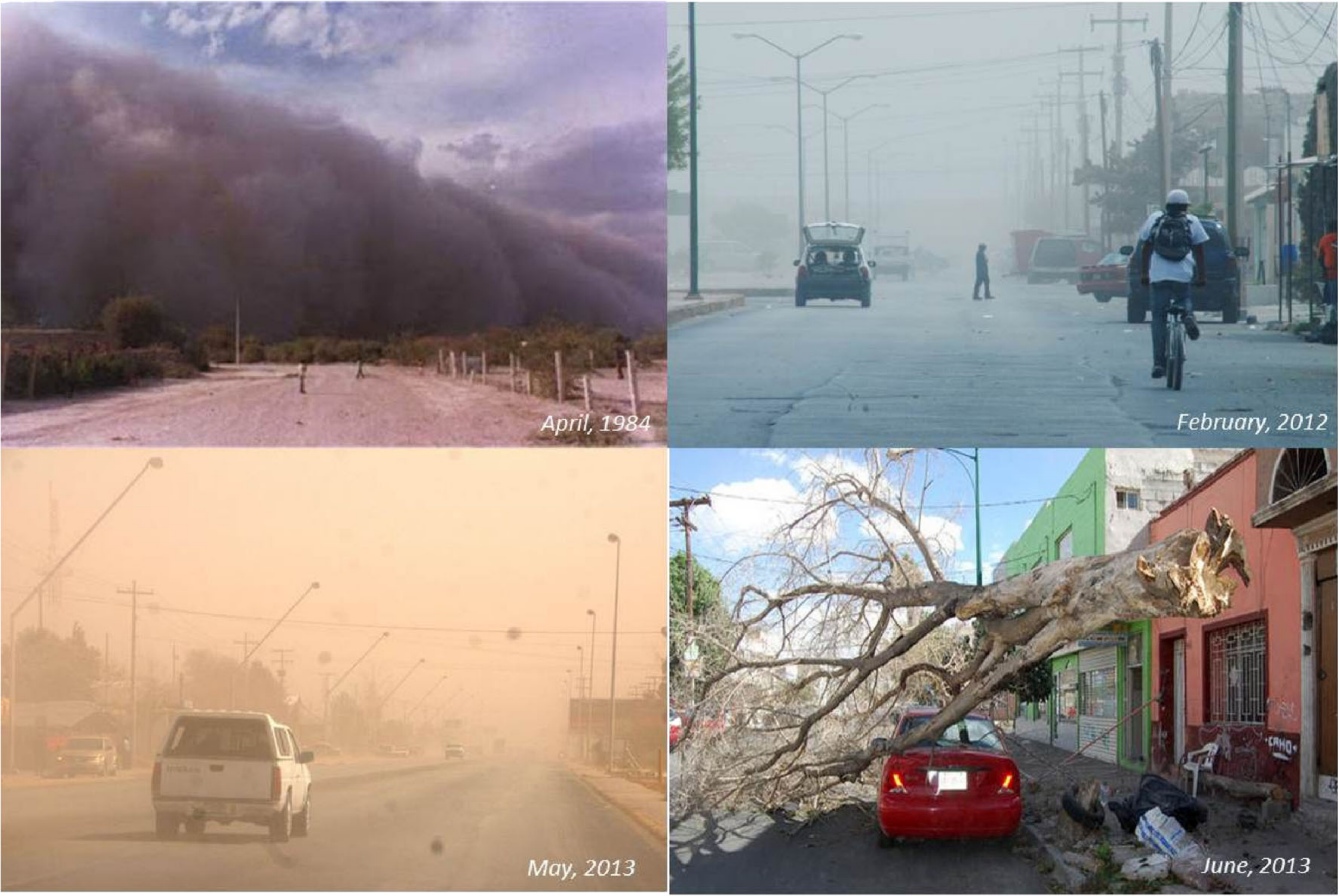
Dust storms that occurred at different times in rural and urban areas of the municipality of Lerdo

This decrease in humidity, as shown in Fig. 8, could not only negatively affect rates of soil erosion, with soil losses of up to 147.2 t ha^−1^ yr^−1^ likely in the future under ScA2, but also lead to changes in the biofeedback systems (D’Odorico et al. 2013; Harper et al. 2010), crises in terms of the availability of fresh water and negative effects on air quality associated with a drastic increase in particulate matter (Singh et al. 2012), and increasingly dangerous levels of lead (Pb) (Garcia-Vargas et al. 2014), calcium (Ca^2+^), and arsenic (As), which have been reported in similar environments (Brahney et al. 2013). These changes are potentially damaging to the health of individuals (Almasi et al. 2014; Razo et al. 2004) and could affect the albedos of the Earth’s surface and atmosphere (Batjargal et al. 2006; Kim et al. 2011). Figure 9 shows critical times of drought impacts, which are generally poorly documented (Martínez et al. 2000) despite the existence of an environmental monitoring network for air quality that includes the neighboring municipalities of Lerdo and Gomez Palacio, Durango (SEMARNAT 2010).

The laminar wind erosion rate calculated for ScA2 (16.8–147.2 t ha^−1^ year^−1^) would cause the removal and transport of a soil layer between 1.68 and 14.7 mm thick. The majority of the territory is characterized by soil with a medium texture, and the bulk density ranges from 1,100 to 1,200 kg m^−3^, which implies a potential loss of the soil layer up to 0.5 m thick over nearly 30 years. For this soil type, these values exceed the rates of soil loss tolerance (5–12 t ha^−1^ year^−1^) proposed by the Department of Agriculture of the United States of America [USDA, (Montgomery 2007)]. The level of environmental degradation resulting from climate variability and poor practices in the management of natural resources affected a global total of 36 × 10^8^ ha by early 1990, 15.2 % of which corresponds to the impacts of wind erosion (Stavi and Lal 2014). The aridity trend observed in the present study (Fig. 9b) is more likely to dramatically impact the population of the slightly over 213,000 inhabitants who are estimated to reside in the Lerdo municipality in 2039 (SEMARNAT 2013).

In addition to the municipal seat, other seats that expect trends of increasing population growth, according to the Study of Ecological Planning and Land Survey Township Lerdo, Durango Planning (SEMARNAT 2013), are located in areas classified with a high index of aridity: Villa de Guadalupe and the Huarache (Guarache), followed by the city of Lerdo, La Loma Nazarene, and Ciudad Juárez. Conversely, the seats that will have small significant increases in the coming years will be Álvaro Obregón, Leon Guzman, Juan E Garcia, and Sapioris.

## Conclusions

The results for the two EQI examined, LWER and AI, suggest that in the near future (2010–2039), the conditions of environmental stress in the town of Lerdo could increase, with direct impacts on water availability, affecting both economic activities (primarily agriculture) and domestic consumption. Higher temperatures during drought and low rainfall in summer could result in more frequent dust storms with higher particle densities. Hence, the risk to a greater number of people in the southeast area of the municipality would increase.

Finally, the magnitude and distribution of the incidence of impacts in the territory are relevant to the planning and management of natural resources. This research should be considered when making decisions concerning the prevention of impacts of climate on natural resources and public health, both now and in the future.

## Abbreviations

AI: Aridity index (dimensionless)
CATEX: Index related to textural class
CAUSO: Index related to land use
SELM: Studies of ecological land management
EQI: Environmental quality index or indicators
GHGs: Greenhouse gases
GROPE: Number of days per year with suitable water availability and favorable conditions for the establishment of a crop
GROPEA2: GROPE for scenario A2
GCM: Global Circulation Models
HGROPE: Historic GROPE
HLWER: Historic LWER
HMAR: Historic mean annual rain
HMAT: Historic mean annual temperature
IAVIE: Index of wind aggressiveness
INECC: National Institute of Ecology and Climatic Change (prior to 2012, it was INE; it is a division of the Ministry of Natural Resources and Environment)
IPCC: Intergovernmental Panel on Climate Change
LUV: Land use and vegetation
LWER: Laminar wind erosion rate (t ha^−1^ year^−1^)
LWERA2: LWER for scenario A2
MAR: Mean annual rain
MAT: Mean annual temperature
ScA2: Scenario A2
SE: Socio-environmental
WS: Weather stations
WS-NWS: Weather stations of the National Weather Service

## References

[CR1] Al-Kaisi MM, Elmore RW, Guzman JG, Hanna HM, Hart CE, Helmers MJ, Hodgson EW, Lenssen AW, Mallarino AP, Robertson AE, Sawyer JE (2012). Drought impact on crop production and the soil environment: 2012 experiences from Iowa. J Soil Water Conserv.

[CR2] Almasi A, Mousavi AR, Bakhshi S, Namdari F (2014) Dust storms and environmental health impacts: 353–356

[CR3] Batjargal Z, Dulam J, Chung YS (2006). Dust storms are an indication of an unhealthy environment in East Asia. Environ Monit Assess.

[CR4] Brahney J, Ballantyne AP, Sievers C, Neff JC (2013). Increasing Ca^2+^ deposition in the western US: the role of mineral aerosols. Aeolian Res.

[CR5] Buchholz S, Krein A, Junk J, Heinemann G (2014). Size-segregated atmospheric particle mass concentration in urban areas in Luxembourg. Water Air Soil Pollut.

[CR6] Conde C, F Estrada, B Martínez, O Sánchez, Gay C (2011) Regional climate change scenarios for México. Atmósfera 24(1): 125–140. Available from: http://www.journals.unam.mx/index.php/atm/article/view/23806

[CR7] D’Odorico P, Bhattachan A, Davis KF, Ravi S, Runyan CW (2013). Global desertification: drivers and feedbacks. Adv Water Resour.

[CR8] DOF (2012) Ley General de Cambio Climático. Diario Oficial de la Federación, México, D.F. Available from: http://www.diputados.gob.mx/LeyesBiblio/pdf/LGCC.pdf

[CR9] FAO-ISRIC-ISSS (1998). World reference base for soil resources. World Soil Resources Reports 84.

[CR10] García-Páez F, Cruz-Medina IR (2009). Variabilidad de la precipitación pluvial en la región Pacífico norte de México. Agrociencia.

[CR11] Garcia-Vargas G, SJ Rothenberg, EK Silbergeld, V Weaver, R Zamoiski, C Resnick, M Rubio-Andrade, PJ Parsons, AJ Steuerwald, A Navas-Acién, Guallar E (2014) Spatial clustering of toxic trace elements in adolescents around the Torreón, Mexico lead–zinc smelter. Journal of Exposure Science and Environmental Epidemiology. Feb:1–9 doi:10.1038/jes.2014.11 10.1038/jes.2014.11 PMC4737620 24549228

[CR12] González MG (2012) Las zonas áridas y semiáridas de México, y su vegetación. 1st edn. Ed. SEMARNAT-INECC. Impreso en México, 194 P. ISBN 978-607-7908-69-2

[CR13] Harlan SL, Chowell G, Yang S, Petitti DB, Butler EJM, Ruddell BL, Ruddell DM (2014). Heat-related deaths in hot cities: estimates of human tolerance to high temperature thresholds. Int J Environ Res Public Health.

[CR14] Harper RJ, Gilkes RJ, Hill MJ, Carter DJ (2010). Wind erosion and soil carbon dynamics in south-western. Aust Aeolian Res.

[CR15] Howell L, C Hayashi, Zwahlen C et al. (2013) Global risks 2013. World Economic Forum, Geneva Switzerland. (ISBN: 92-95044-50-9). Available from: http://reports.weforum.org/global-risks-2013/

[CR16] INECC-SEMARNAT (2012) México, quinta comunicación nacional ante la Convención Marco de las Naciones Unidas sobre el Cambio Climático (ISBN: 978-6078246-50-2) vol 1. SEMARNAT-INECC, México, D.F. 442 p. Available from: http://www2.inecc.gob.mx/publicaciones/consultaPublicacion.html?id_pub=685

[CR17] INEGI (2007a) Conjunto de datos vectoriales de Edafología, Primera ed. INEGI, Aguascalientes, Ags. Mex

[CR18] INEGI (2007b) Conjunto de datos vectoriales de la serie 4 de uso del suelo y vegetación. INEGI, Aguascalientes, Ags. Méx

[CR19] INEGI (2013) México en cifras, información nacional por entidad federativa y municipios. INEGI, Aguascalientes, Ags., Méx

[CR20] IPCC (2007) Contribution of Working Groups I, II and III to the Fourth Assessment Report of the Intergovernmental Panel on Climate Change. Core Writing Team, Pachauri, R.K. and Reisinger, A. (Eds.). IPCC, Geneva, Switzerland. pp 104

[CR21] Field CB, Barros V, Stocker TF, Qin D, Dokken DJ, Ebi KL, Mastrandrea MD, Mach KJ, Plattner G-K, Allen SK, Tignor M, Midgley PM, IPCC (2012). Managing the risks of extreme events and disasters to advance climate change. A special report of working groups I and II of the Intergovernmental Panel on Climate Change.

[CR22] Karaca F (2012). Determination of air quality zones in Turkey JAPCA. J Air Waste Manage Assoc.

[CR23] Kim H-S, Chung Y-S, Lee S-G (2011). Characteristics of aerosol types during large-scale transport of air pollution over the Yellow Sea region and at Cheongwon, Korea, in 2008. Environ Monit Assess.

[CR24] Lim B, Burton I, Malone E, Huq S (2005). Adaptation policy frameworks for climate change: development strategies, policies and measures.

[CR25] López-Santos A, Pinto-Espinoza J, Ramírez-López EM, Martínez-Prado MA (2013). Modeling the potential impact of climate change in northern Mexico using two environmental indicators. Atmosfera.

[CR26] Magaña VO, Zermeño D, Neri C (2012). Climate change scenarios and potential impacts on water availability in northern Mexico. Clim Res.

[CR27] Magaña VO, Caetano E (2007) Pronóstico climático estacional regionalizado para la República Mexicana como elemento para la reducción de riesgo, para la identificación de opciones de adaptación al cambio climático y para la alimentación del sistema: cambio climático por estado y por sector (informe final) vol 1, 1st edn. SEMARNAT-INE, México

[CR28] Martínez OVA, Rincon CBC, Velazco VMR, Lazo JGS, López CC, Cano PR (2000). Asma y medioambiente en la Comarca Lagunera. Rev Alerg Méx.

[CR29] Monterroso AI, Conde AC, Rosales D, Gómez JD, Gay C (2011). Assessing current and potential rainfed maize suitability under climate change scenarios in Mexico. Atmosfera.

[CR30] Montgomery DR (2007). Soil erosion and agricultural sustainability. PNAS.

[CR31] Moss RH, Edmonds J, Hibbard KA (2010). The next generation of scenarios for climate change research and assessment. NATURE.

[CR32] Ortiz-Solorio CA (1987). Elementos de agrometeorologia cuantitativa con aplicaciones en la Rep. Mexicana.

[CR33] Poulsen L (2013) Costs and benefits of policies and practices addressing land degradation and drought in the drylands. White Paper II. UNCCD 2nd Scientific Conference. UNCCD Secretariat, Bonn. Available from http://2sc.unccd.int (accessed 26 March 2013)

[CR34] Razo I, Carrizales L, Castro J, Díaz-Barriga F, Monroy M (2004). Arsenic and heavy metal pollution of soil, water and sediments in a semi-arid climate mining area in Mexico. Wate Air Soil Pollut.

[CR35] Rivera DR, Crespo RGP, Arteaga RR, Quevedo AN (2007). Comportamiento espacio temporal de la sequía en el estado de Durango. Méx Terra Latinoam.

[CR36] Salinas-Zavala CA, Llich-Belda D, Hernández-Vázquez S, Lluch-Cota BD (1998). La aridez en el noroeste de México. Un análisis de su variabilidad espacial y temporal. Atmósfeta.

[CR37] SEDESOL-INE (1989). Ordenamiento Ecológico del territorio.

[CR38] SEMARNAT (2010). Programa para Mejorar la Calidad del Aire en la Región de la Comarca Lagunera.

[CR39] SEMARNAT (2013). Estudio técnico para el ordenamiento ecológico y territorial del municipio de Lerdo, Durango.

[CR40] SEMARNAT-INECC (2011) Sistema de información de escenarios de cambio climático regionalizados (SIECCRe), INECC. http://zimbra.ine.gob.mx/escenarios. Accessed Sep 2011

[CR41] Singh P, Sharratt B, Schillinger WF (2012). Wind erosion and PM10 emission affected by tillage systems in the world’s driest rainfed wheat region. Soil Till Res.

[CR42] Stavi I, Lal R (2014) Achieving zero net land degradation: challenges and opportunities. J. of Arid Environments 1–8. doi:10.1016/j.jaridenv.2014.01.016

[CR43] Zhang Q, Li J, Singh VP, Xu CY, Bai Y (2012). Changing structure of the precipitation process during 1960–2005 in Xinjiang, China. Theor Appl Climatol.

[CR44] Zhang Z, Dong Z, Chen S (2013). Wind erodibility in eastern Ningxia Province, China. Environ Earth Sci.

